# Long-term high-fat diet and long-term high-refined-carbohydrate diet induce alterations in the splenic *TCRβ CDR3* repertoire and peripheral multi-compartmental immune parameters in mice

**DOI:** 10.3389/fimmu.2026.1947535

**Published:** 2026-10-14

**Authors:** Jiayi Wang, Shoupan Gao, Yuchun Cai, Yating Zhang, Wenci Weng, Pengxun Han, Huili Sun

**Affiliations:** Department of Nephrology, Shenzhen Traditional Chinese Medicine Hospital, The Fourth Clinical Medical College of Guangzhou University of Chinese Medicine, Shenzhen, Guangdong, China

**Keywords:** CDR3, high refined carbohydrate diet, high-fat diet, next-generation sequencing, TCR

## Abstract

**Background:**

High-fat diet (HFD) and high-refined carbohydrate diet (HRCD) are closely associated with elevated risks of chronic inflammation, autoimmune diseases, and infectious diseases. However, the molecular alterations of the immune system following long-term intervention with these two diets remain unclear.

**Methods:**

3-month-old young male mice were used as the animal model and received long-term intervention with HFD or HRCD until 14 months of age. Comparative analyses were performed on the pathological characteristics of immune organs, immune cell subsets, serum proteomics, and *TCRβ CDR3* repertoire characteristics detected by next-generation sequencing.

**Results:**

Both dietary interventions led to increased body weight and caused similar splenic pathological damage in mice. Their effects on the immune system were dominated by peripheral alterations with mild central changes. Meanwhile, both diets led to increased clonal concentration, decreased uniformity, depletion of low-frequency clones, and expansion of mid-frequency clones in the splenic *TCRβ CDR3* repertoire while also identifying a clonotype CSSRAGGLNSDYTF whose abundance in both the thymus and spleen was significantly higher in the HFD and HRCD groups than in the Control group. In contrast, the specific mechanisms underlying immune regulation differed significantly between the two diets. HFD significantly upregulated the levels of serum IL-6, IL-10, TNF-α and FAS, altered the proportion of splenic T cell subsets, and strongly affected splenic *TRBJ* gene usage and CDR3 length distribution. HRCD predominantly increased IL-17A levels and the proportion of DCs.

**Conclusion:**

This study preliminarily demonstrates that HFD and HRCD can induce similar pathological phenotypes but distinct molecular immune mechanisms, both showing more pronounced alterations in the spleen than in the thymus under the parameters examined.

## 1 Introduction

Nowadays, metabolic homeostasis disruption induced by long-term high-fat diet (HFD) and high-refined carbohydrate diet (HRCD) has become a high-risk factor contributing to systemic metabolic disorders and impaired immune function (1). Metabolic disorders driven by different dietary patterns increase the risk of chronic low-grade inflammation (2), autoimmune diseases (3), and infectious diseases (4), while these changes act synergistically with aging to accelerate the process of immune senescence (5).

Studies have confirmed that the impacts of HFD on the immune system include inducing splenic oxidative stress and significantly promoting lymphocyte apoptosis in the spleen (6). Meanwhile, by upregulating the levels of pro-inflammatory cytokines such as IFN-γ, granzyme B and GM-CSF, HFD disrupts the balance of T cell subsets, induces the expansion of effector memory T cells and the reduction of naive T cells in the spleen and adipose tissue, and is characterized by abnormally elevated proportions of CD4^+^ and CD8^+^ T cells in adipose tissue (7). Following feeding of mice with a high-refined-carbohydrate diet, infiltration of macrophages, T cells (CD8^+^ and CD4^+^Foxp3^+^ subsets), and neutrophils was observed in adipose tissue, accompanied by persistently elevated serum levels of cytokines including TNF-α, IL-6, and IL-10 (8). Meanwhile, high-sucrose diets can trigger intestinal microbiota dysbiosis, which reduces the abundance of intestinal protective Th17 cells, disturbs intestinal immune-cell subset composition, and up-regulates the expression of inflammatory cytokines (9). These alterations further provoke adipose-tissue inflammation and increase pro-inflammatory factor levels in adipose tissue (9, 10). Global deficiency of cDC1 in Batf3^–^/^–^ mice is also associated with exacerbated hepatic inflammation under high-sucrose dietary challenge (11). In addition, high-glucose intake promotes the differentiation of pathogenic Th17 cells via mitochondrial reactive oxygen species (12). Four-week high-fructose exposure enhances small-intestinal epithelial-cell survival, lengthens intestinal villi, and enlarges the intestinal nutrient-absorptive surface area (13). By contrast, 8-week high-fructose consumption specifically drives Th1/Th17 differentiation and markedly aggravates intestinal inflammation through glutamine-metabolism-dependent mTORC1 activation as well as reactive-oxygen-species-mediated TGF-β activation (14). Nevertheless, studies regarding the effects of HRCD on central and peripheral immune organs remain absent to date.

The diversity and characteristics of the T cell receptor beta chain complementarity- determining region 3 (*TCRβ CDR3*) repertoire are key indicators reflecting the specificity of immune responses, and alterations in these characteristics are a direct reflection of the immune functional status of the organism (15, 16). Current studies on the impacts of high-energy diets on the TCR repertoire have mostly focused on HFD, which has been proven to induce a reduction in *TCRβ CDR3* diversity in adipose tissue, accompanied by altered *V* gene usage preferences, increased TCR clonality in CD8^+^ T cells and changes in CDR3 length distribution (7). However, the alterations in *TCRβ CDR3* repertoire characteristics induced by HRCD remain unclear. In addition, current studies related to HFD and HRCD still have obvious limitations: the intervention period is generally short, and such long-term models with continuous intervention from adulthood to late-middle age (11 months) remain scarce; the influences of HFD and HRCD on the TCR repertoire in central (thymus) and peripheral (spleen) immune organs still lack comprehensive and systematic analyses; and comparative studies on their differential impacts and underlying mechanisms remain unknown.

Accordingly, 3-month-old mice were used as research subjects in the present study and fed with HFD or HRCD until 14 months of age. We systematically analyzed the proportions of immune cell subsets and comprehensively characterized the *TCRβ CDR3* repertoire features in the thymus and spleen under these two dietary stresses. This study aimed to elucidate the differential regulatory patterns of the long-term high-energy diets on the immune system and screen for potential high-energy diet-sensitive CDR3 signature clonotypes.

## 2 Materials and methods

### 2.1 Ethics

All procedures were performed accordance with the Guide for the Care and Use of Laboratory Animals. All procedures were approved by the Animal Ethics Committee (IACUC) of PKUSZ (approval no.AP0044004). Every effort was made to minimize animal suffering and distress throughout the experiments.

### 2.2 Laboratory animal

Twenty-four male C57BL/6J mice were purchased from Gempharmatech Company (Guangdong, China) and randomly divided into three groups (n=8 per group): the high-fat diet (HFD) group, the high-refined carbohydrate diet (HRCD) group, and the control group. All mice were housed under specific pathogen-free (SPF) conditions with a 12-hour light/dark cycle, constant temperature (22 ± 2°C) and relative humidity (50 ± 10%). All mice had free access to food and water. After 1 week of acclimatization, mice were maintained until they reached 3 months of age. The HFD group was fed a high-fat diet which consisted of 60% fat (mainly lard and soybean oil), 20% protein, and 20% carbohydrates (Research Diets Inc., NJ, USA). The HRCD group received a high-refined carbohydrate diet which consisted of 70% refined carbohydrates (corn starch, maltodextrin, sucrose and other refined carbohydrates), 20% protein, and 10% fat (Research Diets Inc., NJ, USA). The control group was fed a standard chow which consisted of 63% carbohydrates (mainly corn and other crude carbohydrates), 13% fat and 24% protein (Beijing Keao Xieli Feed Co., Ltd, Beijing, China). All mice were maintained on these diets for 11 months, up to 14 months of age. Food consumption was monitored regularly to calculate caloric intake. At the endpoint, mice were weighed and anesthetized with inhaled isoflurane (~3% for induction and 1–2% for maintenance), and then all mice were euthanized together by cervical dislocation. Death was confirmed by cessation of heartbeat and respiratory arrest. Spleens were weighed and sectioned for staining; thymi and spleens were collected for flow cytometry detection and DNA extraction. Serum samples were isolated, aliquots of which were subjected to measurements of serum Triglyceride (TG) and Total Cholesterol (TC) using an automatic biochemical analyzer (Roche, Basel, Switzerland), as well as Fasting Blood Glucose (FBG) determination via a blood glucose meter (Roche, Basel, Switzerland). The remaining serum was used for Olink proteomics detection.

### 2.3 Hematoxylin-eosin staining

Paraffin-embedded spleen sections from mice in the three groups were stained using an H&E kit (Solarbio, Beijing, China) according to the manufacturer’s instructions. After dehydration, clearing, and mounting with neutral balsam, the sections were scanned with the Digital Slide scanner (3DHistech Ltd., Budapest, Hungary). Spleens from three mice were used as biological replicates (n=3); one H&E-stained section was prepared per spleen. Sections were imaged at 5× magnification, with 2–3 representative fields captured per section. In ImageJ, white-pulp was manually outlined in each field. Red-pulp area was calculated as total tissue area minus white-pulp area. For each mouse, the summed red-pulp area across 2–3 fields was divided by the summed white-pulp area from the same fields to yield the red-to-white-pulp ratio. All histological imaging and area quantification were performed under single-blind conditions. Random sample IDs were used throughout the assay. After all image analysis was completed, the principal investigator unblinded group information for statistical analysis to reduce observer bias.

### 2.4 Immunohistochemical staining

Mouse spleen tissues were fixed, embedded in paraffin, and serially sectioned at a thickness of 4 μm. Sections were deparaffinized twice in fresh xylene, rehydrated through graded ethanol series, and subjected to citrate buffer mediated antigen retrieval. Sections were incubated overnight at 4°C with primary antibody against CD3 (Proteintech, IL, USA), washed, and then incubated with ready to use horseradish peroxidase conjugated secondary antibody (Vazyme Biotech Co., Ltd., Nanjing, China) without dilution at room temperature. Sections were visualized with 3, 3’ diaminobenzidine and counterstained with hematoxylin. For quantitative analysis, sections were imaged at 10× magnification. In ImageJ, total splenic white-pulp areas were manually outlined. Manually delineated periarteriolar lymphoid sheath (PALS) regions with densely distributed CD3 positive cells were defined as the CD3^+^ T cell zone. CD3^+^ T cell zone area proportion was calculated as CD3^+^ T cell zone area/total white-pulp area, expressed as percentage. Spleens from three mice per group served as biological replicates. Multiple white-pulp regions were analyzed for each mouse, and individual mean values were used for statistics. All histological imaging and area quantification were performed under single-blind conditions as H&E staining.

### 2.5 Flow cytometry

Spleen and thymus tissues were minced with scissors and gently homogenized using a syringe plunger. The resulting cell suspension was filtered through a 70 μm cell strainer, collected in a centrifuge tube, and washed with PBS to obtain a single-cell suspension. After staining with a fixable viability dye for 10 minutes, the cells were incubated with an Fc-receptor-blocking antibody (anti-CD16/CD32) for 10 minutes. Thymocytes were stained with antibodies against CD3ϵ, CD4, CD8, CD44, and CD62L for 30 minutes; splenocytes were stained with antibodies against CD3ϵ, CD4, CD8, NK1.1, B220, CD11c, and MHC class II for 30 minutes. Subsequently, the prepared single-cell suspensions were acquired and analyzed on a BD FACSAria III flow cytometer (BD Biosciences, NJ, USA). All antibodies and the viability dye were also purchased from BD Biosciences.

### 2.6 Olink proteomic analysis

The serum proteins were measured using the Olink^®^ Target 96 Mouse Exploratory panel (Olink Proteomics AB, Uppsala, Sweden) according to the manufacturer’s instructions. The final assay read-out is presented in NPX values, which is an arbitrary unit on a log2-scale where a high value corresponds to a higher protein expression. The resulting data were subsequently analyzed statistically.

### 2.7 *TCRβ CDR3* repertoire amplification and purification

Genomic DNA was extracted from mouse thymus and spleen tissues using the DNeasy Blood & Tissue Kit (Qiagen, Hilden, Germany) and quantified with a NanoDrop OneC spectrophotometer (Thermo Fisher Scientific, MA, USA). Using the extracted mouse DNA as the template, the *TCRβ CDR3* repertoire was amplified by multiplex PCR. PCR products were separated by agarose gel electrophoresis, and target bands were excised under UV light with a sterile scalpel and purified using a Gel Extraction Kit (Qiagen, Hilden, Germany).

### 2.8 Next-generation sequencing

Qualified gel-purified samples were sent to BGI Genomics for sequencing. Following sequencing library construction, the TCRβ CDR3 repertoire was subjected to paired-end 150 bp sequencing on the DNBSEQ-T7 platform. Approximately 19.2 million read pairs per sample were obtained (range: 12.6 million-23.6 million), with a sequencing depth ranging from 28800 × to 57600 ×. Raw data were filtered to remove adapter sequences and low-quality reads and then aligned to the corresponding reference genome.

### 2.9 Bioinformatics analysis

Raw *TCRβ CDR3* sequencing data were processed using MiXCR. Sequences that neither started with cysteine (C) nor ended with phenylalanine (F) were filtered out. A clonotype was defined as a unique TCRβ rearrangement (the complete-identical combination of *TRBV* gene, *TRBJ* gene and CDR3 nucleotides). Data visualization was performed using the R package immunarch, along with VDJTools, GraphPad Prism 8, Adobe Photoshop, FlowJo (version 10.8.1) and ImageJ.

### 2.10 Statistical analysis

Statistical analyses were conducted with R (version 4.3.2) and SPSS Statistics 22. Our study focused on comparisons of the HFD group versus the Control group and the HRCD group versus the Control group; therefore, comparisons between the HFD group and the HRCD group were not performed. For data meeting the assumptions of normality and homogeneity of variance, the independent-samples Student’s t-test was applied. For data that violated the normality assumption, the Mann-Whitney U test was employed. Differences were considered statistically significant at p < 0.05, p < 0.01, or p < 0.001.

### 2.11 Analysis of *TCRβ CDR3* clonal overlap

Briefly, clonotypes intersection analysis was performed among 8 samples in each group. Clonotypes shared by all samples within a group were extracted as the core representative clonotypes of that group. Subsequently, pairwise and triple intersection analyses were conducted on the core representative clonotypes across the three groups, and Venn diagrams were constructed based on the number of overlapping clonotypes.

### 2.12 Analysis of differentially abundant CDR3 clonotypes

Core sequences from the HFD and HRCD groups were separately compared with those from the Control group to screen the top significantly differentially abundant clonotypes. The expression frequency of these clonotypes in each sample (8 frequencies per clonotype) was then extracted, subjected to statistical analysis, and visualized.

## 3 Results

### 3.1 HFD and HRCD altered body weight and serum cytokine levels in mice

We regularly recorded and calculated the caloric intake of mice in the three groups. The caloric daily intake was 14.11 ± 0.54 kcal/day for the Control group, 14.40 ± 0.30 kcal/day for the HFD group, and 14.76 ± 0.47 kcal/day for the HRCD group. Following 11-month dietary intervention, mice in the HFD and HRCD groups exhibited markedly increased body size and body weight compared with the Control group (**Figures 1A, B**). The average daily food intake of mice in both the HFD and HRCD groups was significantly lower than that in the Control group (**Figure 1C**), whereas no significant difference was observed in average daily caloric intake (**Figure 1D**). Serum samples from mice were subjected to analyses of TG (**Figure 1E**), TC (**Figure 1F**), as well as proteomic analysis (**Figure 1H**), while FBG (**Figure 1G**) was determined using whole blood. The results showed that there was no significant difference in serum TG among groups. Serum TC and FBG levels were significantly higher in both the HFD and HRCD groups than in the Control group. The protein levels of IL-6, IL-10, TNF-α and FAS were significantly elevated in the HFD group relative to the Control group. Serum protein levels of IL-10, IL-17A, TNF-α and FAS were significantly increased in the HRCD group compared with the Control group.

**Figure 1:**
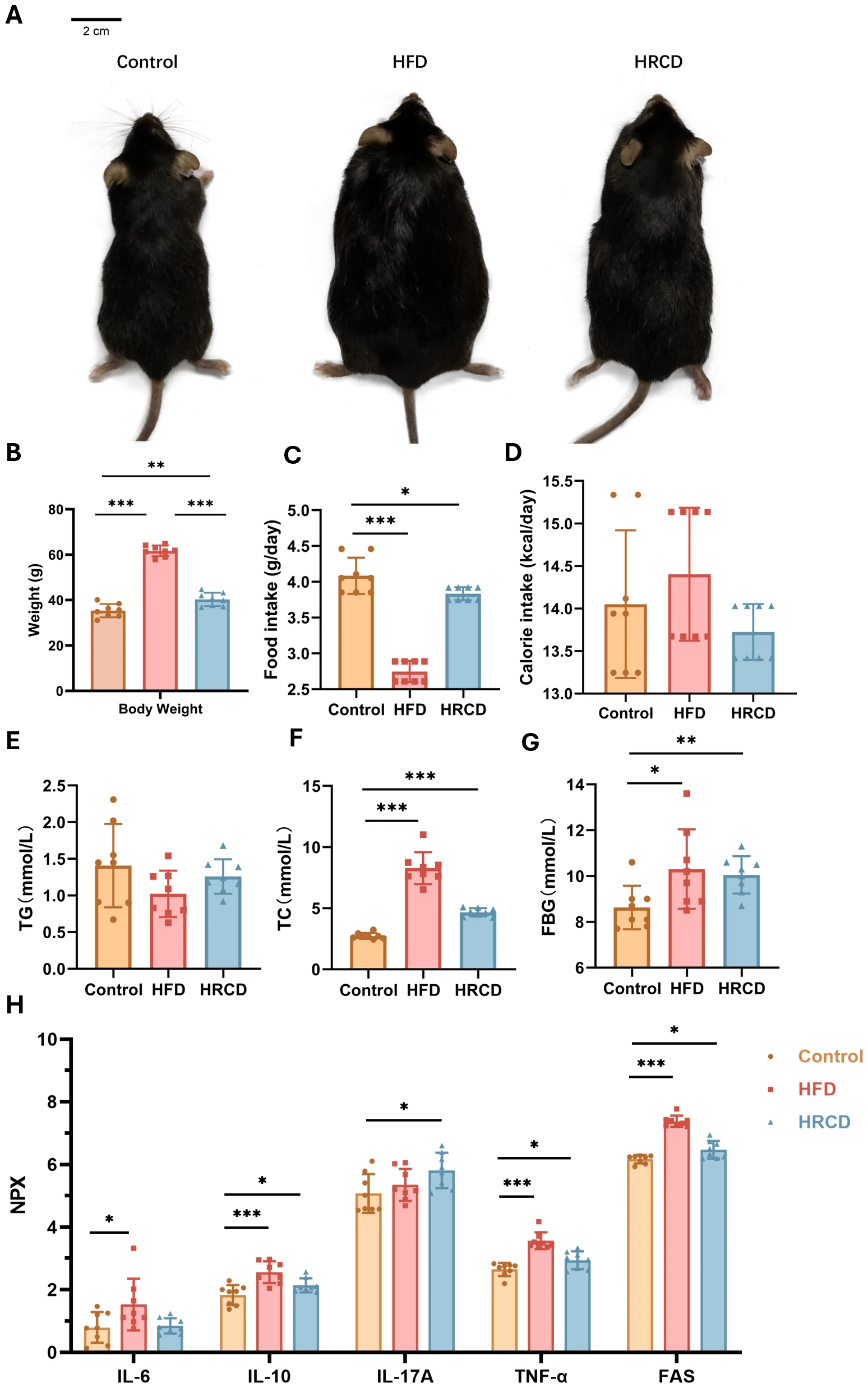
Comparative analysis of gross photographs, body weight, and serum cytokine levels in mice. Representative photographs showing the overall morphology of mice **(A)**, with a scale bar of 2 cm; comparisons of body weight **(B)**, average daily food intake (g/day) **(C)**, average daily caloric intake (kcal/day) **(D)** Triglyceride (TG) **(E)**, Total Cholesterol (TC) **(F)**, Fasting Blood Glucose (FBG) **(G)**, and the levels of various serum immune molecules **(H)** were presented (n=8). *p < 0.05, **p < 0.01, ***p <0.001.

### 3.2 HFD and HRCD altered the proportions of immune cell subsets in the spleens of mice

The differences identified by serum proteomics prompted us to investigate whether there were also alterations in the composition of immune cell populations among the three groups. We then performed flow cytometric analysis to examine the proportions of immune cell subsets in the thymus and spleen of mice from the three groups. We found that no significant changes in any cell subset were observed in the thymus (**Figure 2C**), and the alterations in immune cell proportions appeared to be restricted to the spleen, a major peripheral immune organ. The proportions of total T cells, CD8^+^T cells and NKT cells in the spleen were all significantly reduced in the HFD group compared with the Control group, whereas the HRCD group showed a markedly higher proportion of splenic DC cells relative to the Control group (**Figures 2A, B**).

**Figure 2:**
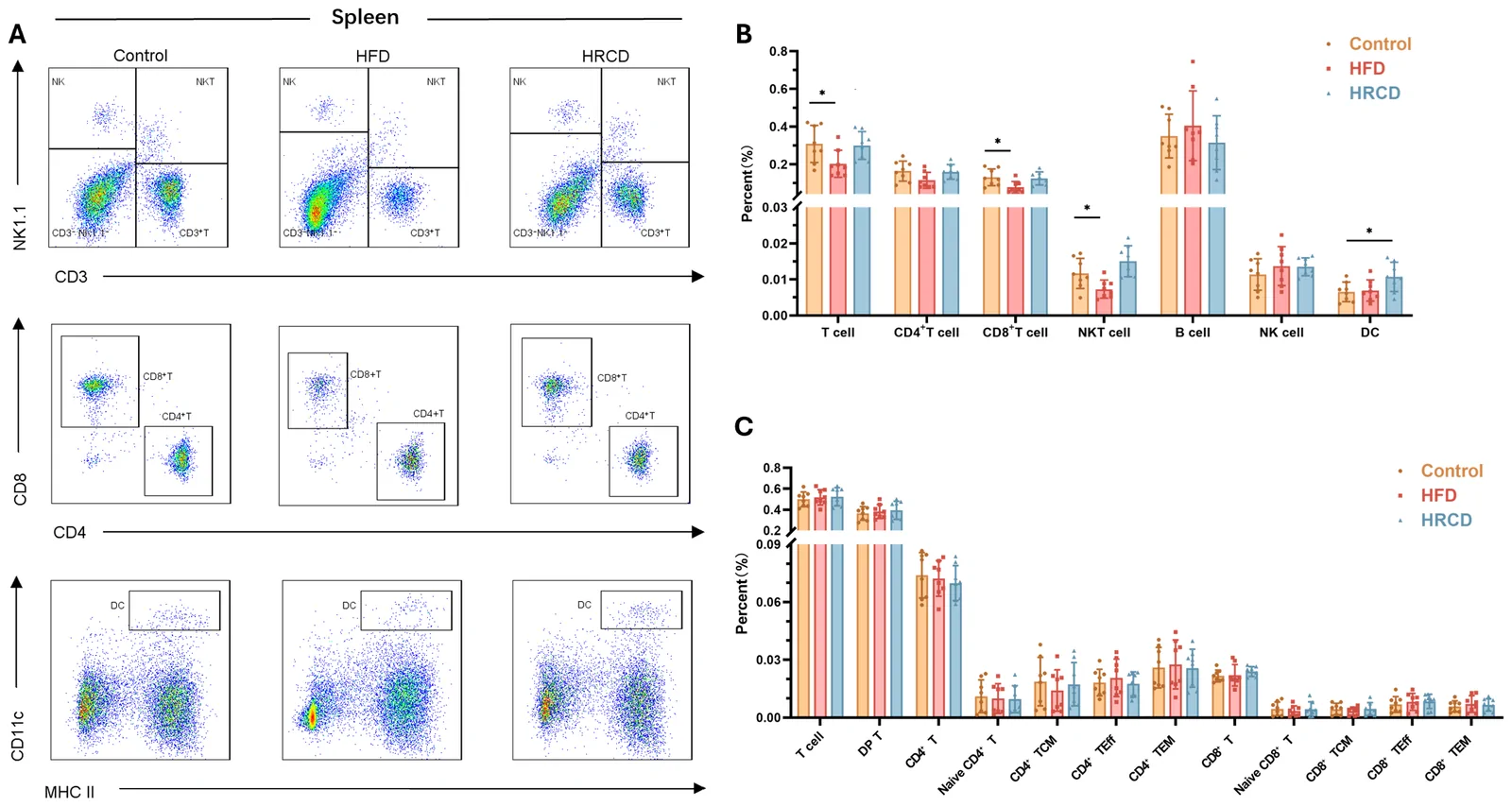
Flow cytometric analysis of lymphocyte subsets in the spleen of mice. Representative flow cytometric plots of the spleen (one sample per group shown as a representative example, with only subsets with statistically significant differences displayed) were shown in **(A)**; corresponding statistical results of splenic immune cell subsets were presented in **(B)**, and those of thymic immune cell subsets were presented in **(C)** (n=8). *p < 0.05.

### 3.3 HFD induced pathological changes in the spleen of mice

Given that the changes observed in flow cytometry were predominantly localized to the spleen, the spleen pathological changes and immune cell distribution were assessed. The results demonstrated a marked increase in spleen weight in the HFD group compared with the Control group, while the HRCD group showed an increasing trend but no statistically significant difference (**Figures 3A, B**). H&E staining revealed that, compared with the Control group, mice in both HFD and HRCD groups exhibited irregular morphology of the white-pulp and blurred junction between the white and red pulp; these alterations were more pronounced in the HFD group than in the HRCD group. Moreover, both the white-pulp proportion and the red-pulp-to-white-pulp ratio (R/W) were significantly elevated in the HFD group, whereas no significant difference was observed in the HRCD group (**Figures 3C, D, F**). Immunohistochemistry showed that there was no statistically significant difference in the proportion of CD3^+^ T-cell zone area within splenic white-pulp among the three groups, although a declining trend was observed in both the HFD and HRCD groups (**Figures 3E, G**).

**Figure 3:**
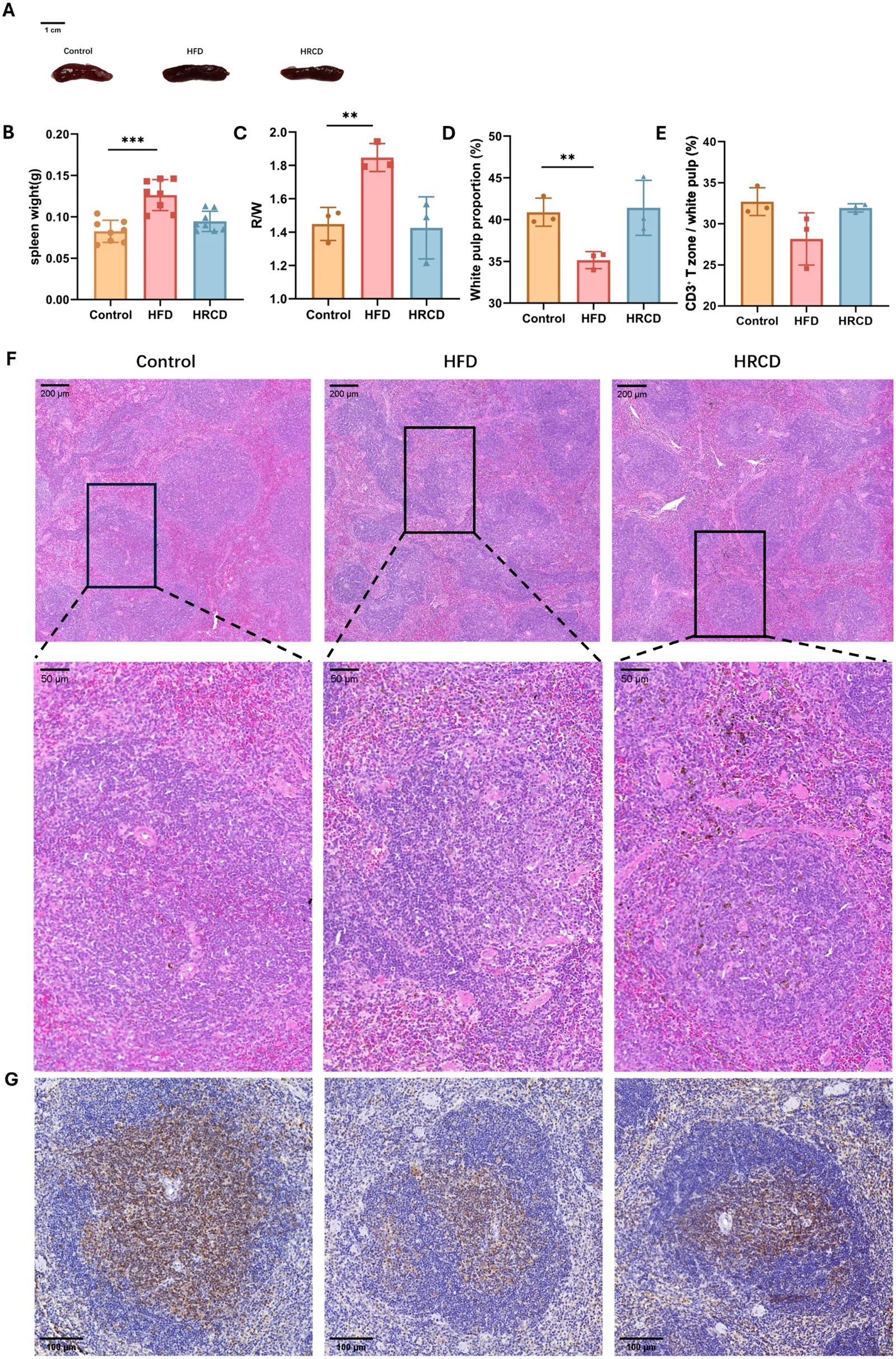
Analysis of spleen morphology, weight, and histological characteristics in mice. Representative photographs of gross spleen morphology **(A)**; spleen weight (n=8) **(B)**; red-pulp-to-white-pulp ratio of the spleen (n=3) **(C)**; white-pulp proportion (n=3) **(D)**; proportion of CD3^+^ T-cell zone area in splenic white-pulp determined by immunohistochemistry (n=3) **(E)**; representative H&E staining images of the spleen **(F)**; representative immunohistochemical images of splenic CD3^+^ T cells **(G)**. **p < 0.01, ***p <0.001.

### 3.4 HFD and HRCD mice exhibited increased clonal convergence and decreased evenness in the splenic *TCRβ CDR3* repertoire

Given that flow cytometric and immune molecular assay results demonstrated that alterations to the murine immune system induced by HFD and HRCD appeared to be predominantly at the T cell level, we performed multiplex PCR amplification of the *TCRβ CDR3* repertoire followed by next-generation sequencing using genomic DNA from the thymus and spleen of mice as templates to investigate the corresponding gene-level changes. In the comparison of *TCRβ CDR3* diversity, no significant differences were found in the thymic D50 diversity index (**Figure 4A**) and inverse Simpson index (**Figure 4B**) between HFD and HRCD mice; in the spleen, the D50 diversity index was found to be significantly decreased in the HFD group compared with the Control group (**Figure 4C**), while no significant difference was observed in the HRCD group relative to the Control group; the inverse Simpson index (**Figure 4D**) showed no statistical significance between the two diet groups and the Control group due to the large standard deviation, yet a clear decreasing trend was found in both the HFD and HRCD groups.

**Figure 4:**
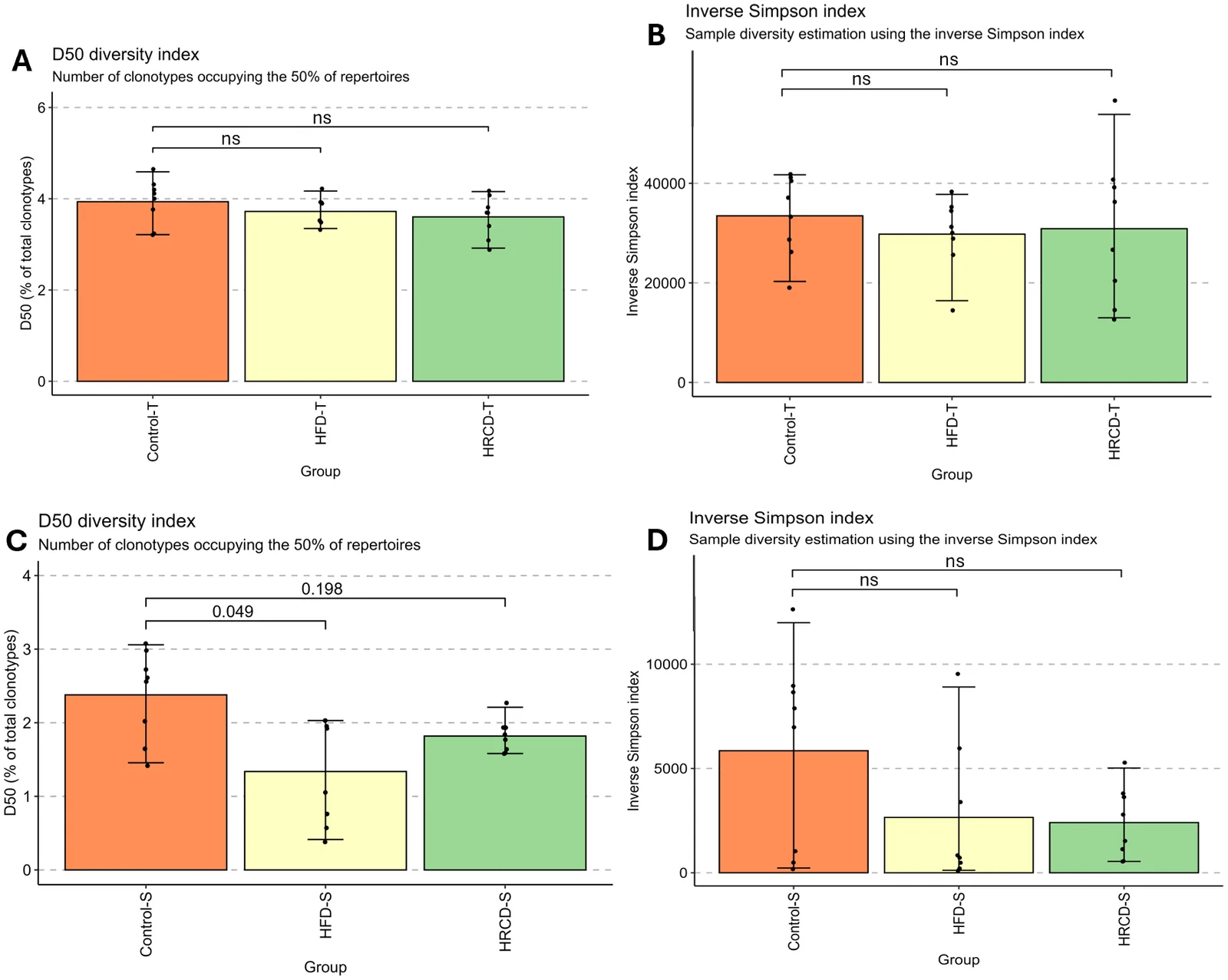
Analysis of *TCRβ CDR3* repertoire diversity in the thymus and spleen of mice. Thymic D50 index **(A)**; Thymic 1/DS index **(B)**; Splenic D50 index **(C)**; Splenic 1/DS index **(D)** (n=8).

### 3.5 HFD and HRCD altered the clonal distribution characteristics of the *TCRβ CDR3* repertoire in the thymus and spleen of mice

To further characterize the specific manifestations of *TCRβ CDR3* repertoire diversity in the thymus and spleen, we analyzed the *TCRβ CDR3* clonotype proliferation in mice under the two dietary interventions. Clonotypes were defined as low (0<x ≤ 10^–4^), medium (10^–4^<x ≤ 0.001), high (0.001<x ≤ 0.01) and ultra-high (0.01<x ≤ 1) frequency clonotypes, respectively. The thymus of HFD mice had significantly lower clonotype numbers at the intervals of 101-2000, 2001–20000 and 5001–50000 compared with the Control group (**Figures 5A–C**). Compared to the Control mice, the clonotype numbers in the spleen of HFD mice were significantly lower at 31-100, 5001–50000 and 50001-70000, but significantly higher at 101-2000 (**Figures 5D, E**). Clonal abundance was lower in the low-frequency clonotype interval and higher in the medium-frequency clonotype interval in the HFD group relative to the Control group (**Figure 5F**). In the comparative analysis of the HRCD group and the Control group, no obvious differences were observed in the thymus (**Figures 5G–I**). However, the spleen of HRCD mice showed significantly lower clonotype numbers at 31–100 and 50001-70000, and significantly higher numbers at 101-2000. Additionally, the clonal abundance in the spleen of the HRCD group was higher than that of the Control group in the medium frequency clonotype interval (**Figures 5J, L**).

**Figure 5:**
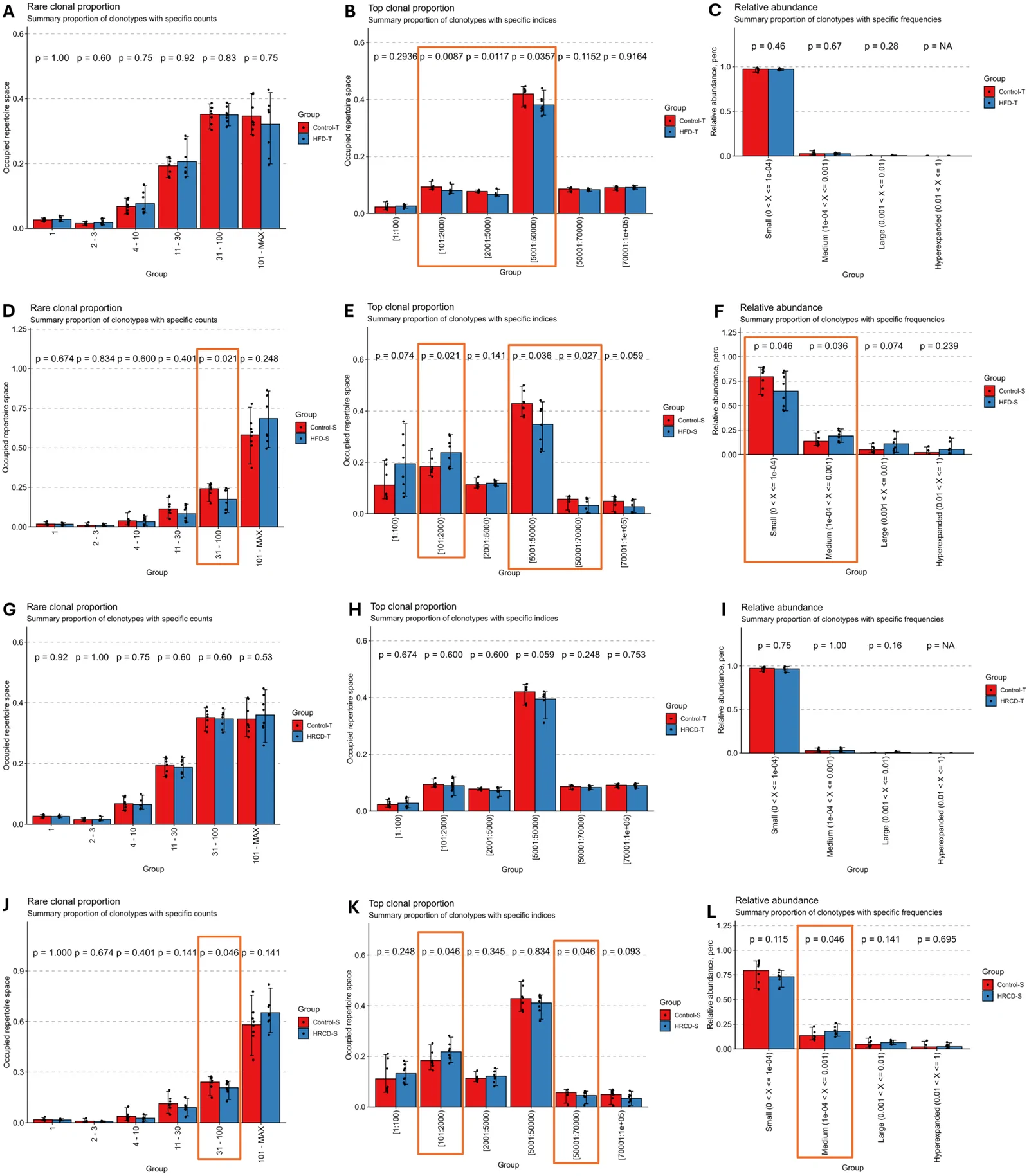
Clonal distribution characteristics of the TCRβ CDR3 repertoire in the thymus and spleen of mice. Comparisons were conducted between the HFD group and Control group in the thymus **(A–C)** and spleen **(D–F)**, as well as between the HRCD group and Control group in the thymus **(G–I)** and spleen **(J–L)**, to illustrate the differential features at the levels of rare clones, top clones, and relative abundance (n=8).

### 3.6 HFD and HRCD had distinct effects on T cell receptor beta variable and joining gene usage in the thymus and spleen of mice

We also analyzed the preferences for *TRBV* and *TRBJ* gene usage induced by the two diets. For the thymus, the HFD group had higher usage of *TRBV12–1* and *TRBV5*, and lower usage of *TRBJ1-5* compared with the Control group (**Figures 6A, B**); For the spleen, the HFD group exhibited higher usage of *TRBV12-1*, *TRBV23*, *TRBJ1–2* and *TRBJ2-1*, and lower usage of *TRBV19*, *TRBV2*, *TRBV4*, *TRBJ2-3*, *TRBJ2-4*, *TRBJ2–5* and *TRBJ2–7* relative to the Control group (**Figures 6C, D**). The thymus of HRCD group had higher usage of *TRBV12-1*, *TRBV31*, *TRBV5* and *TRBJ2-3*, and lower usage of *TRBJ1-1*, *TRBJ1-3* and *TRBJ1-5* compared with the Control group (**Figures 6E, F**); The spleen of the HRCD group had higher usage of *TRBV12–1* and *TRBV12-2*, and lower usage of *TRBV13–1* relative to the Control group, with no significant differences in *TRBJ* gene usage (**Figures 6G, H**). The *TRBV* gene, *TRBJ* gene, and *TRBV-TRBJ* paired usage of individual samples were shown in **Supplementary Figures S1–5**.

**Figure 6:**
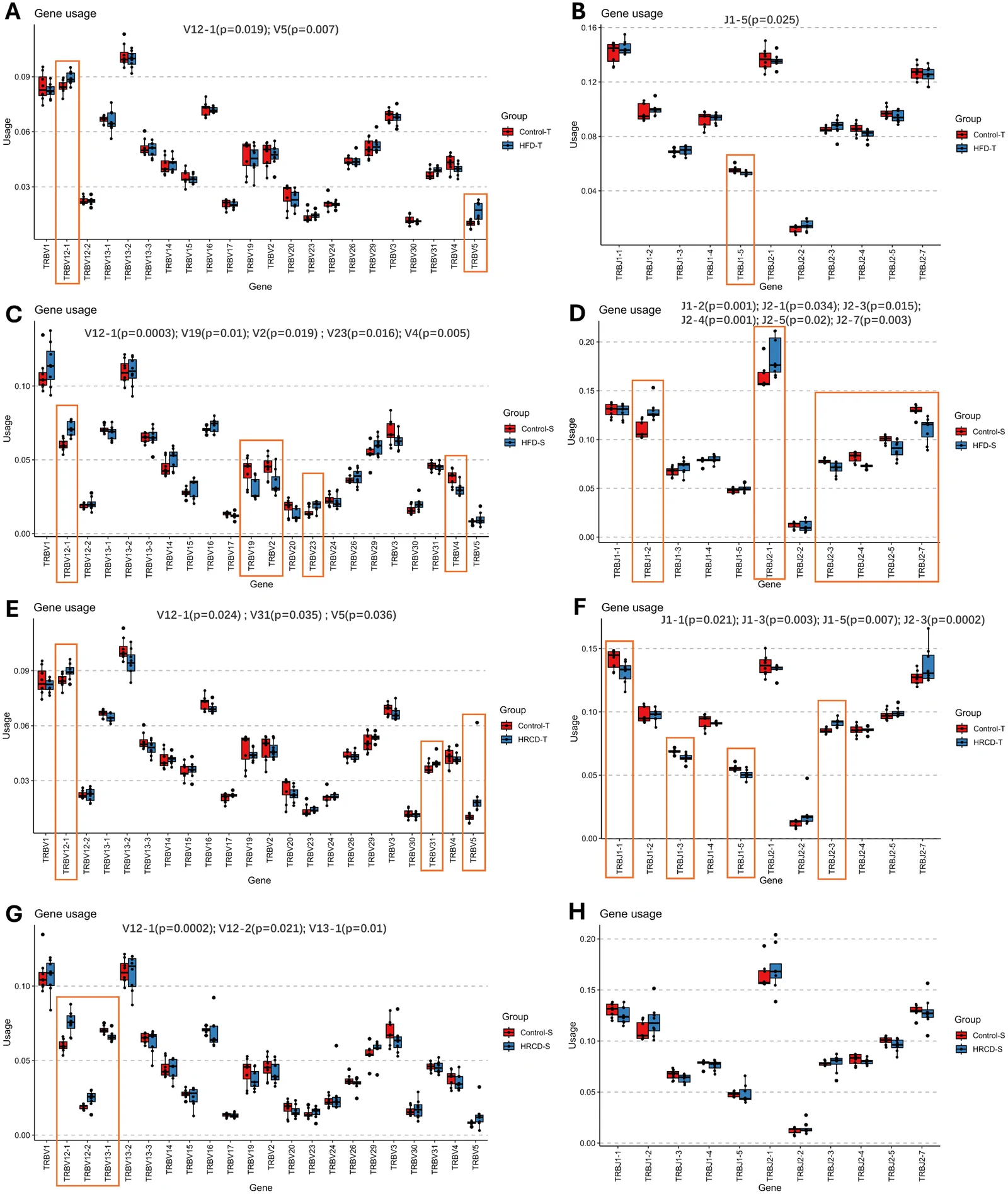
Analysis of TRBV and TRBJ gene usage in the TCRβ CDR3 repertoire in the thymus and spleen of mice. Comparisons of *TRBV* and *TRBJ* gene usage were performed between the HFD group and Control group in the thymus **(A, B)** and spleen **(C, D)**, as well as between the HRCD group and Control group in the thymus **(E, F)** and spleen **(G, H)** (n=8).

### 3.7 HFD and HRCD affected CDR3 length distribution and amino acid usage in the thymus and spleen of mice without shifting the CDR3 length peak

After investigating the differences in *TRBV/TRBJ* gene usage, we further analyzed the CDR3 length distribution and aa usage. No shift in the CDR3 length peak was observed under the two dietary interventions, with the peak still showing a bell-shaped distribution centered on 14-, 15-aa (**Figures 7A, B**). In the thymus, the HFD group had a higher proportion of 11-aa CDR3 compared with the Control group, while the HRCD group had a higher proportion of 8- and 9-aa CDR3 (**Figure 7A**). Analysis of splenic CDR3 length showed that the HFD group had lower proportions of 9-, 12- and 13-aa CDR3 and higher proportions of 17-, 18-, 19-, 25- and >25-aa CDR3 relative to the Control group, while the HRCD group exhibited no significant differences (**Figure 7B**). No significant differences in splenic and thymic aa usage were found among the three groups (**Figures 7C, D**).

**Figure 7:**
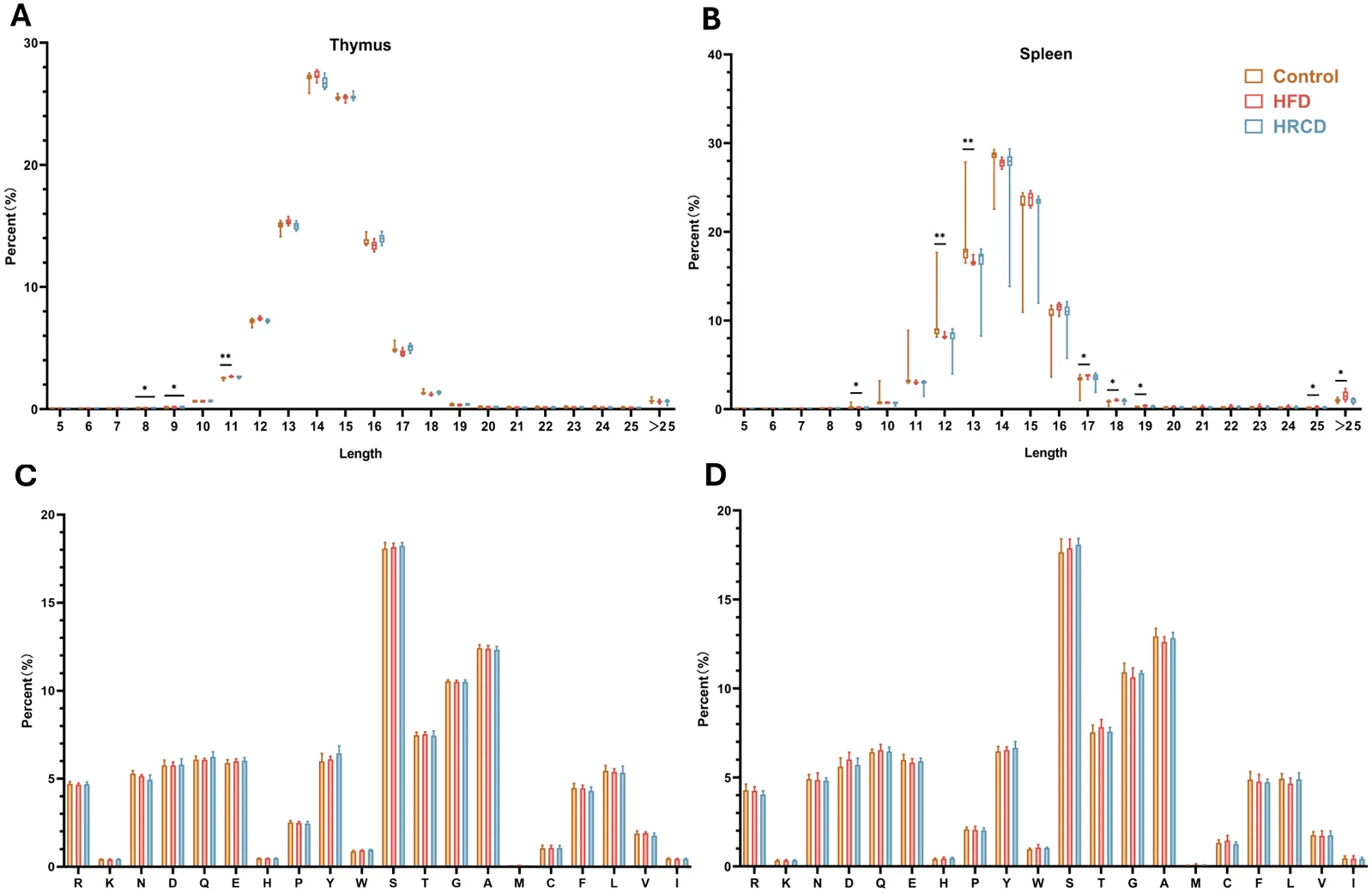
Analysis of *TCRβ CDR3* length and amino acid (aa) usage in the thymus and spleen of mice. Thymic CDR3 length **(A)** and aa usage **(C)** in mice; splenic CDR3 length **(B)** and aa usage **(D)** in mice (n=8). *p < 0.05, **p < 0.01.

### 3.8 HFD and HRCD resulted in high intragroup clonotype similarity in the thymus but marked individual variability in the spleen of mice

Subsequently, we investigated the similarity of the *TCRβ CDR3* repertoire among the three groups by assessing the number of shared clonotypes (reflecting clonotype overlap) and the Jaccard index (quantifying the similarity of clonotype composition). Specifically, the number of shared clonotypes among the three groups in the spleen and thymus is shown in **Figure 8A**. Results of Jaccard index analysis (**Figure 8B**) showed that the Jaccard index of HFD and HRCD mice was higher than that of Control mice in the thymus; no significant differences in the Jaccard index were found between HFD/HRCD and Control mice in the spleen, while the standard deviation of all three groups was increased in the spleen compared with the thymus, especially in the HFD group.

**Figure 8:**
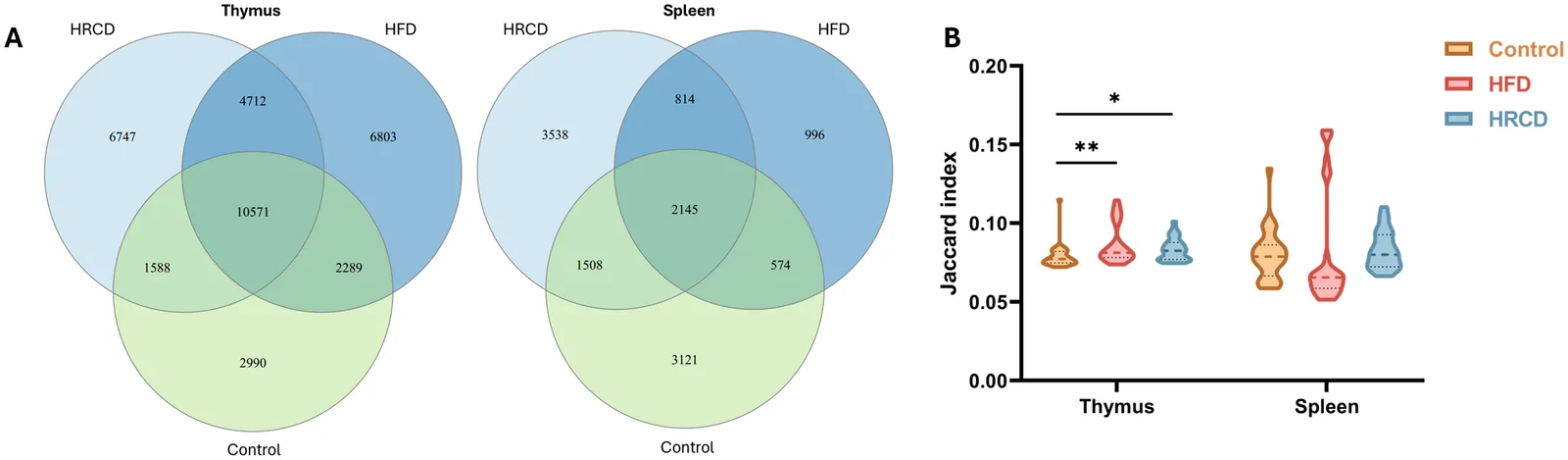
Analysis of *TCRβ CDR3* clonal overlap and similarity in the thymus and spleen of mice. Number of shared clones among the three groups in the thymus and spleen **(A)**; Jaccard index in the thymus and spleen of the three groups **(B)** (n=8). *p < 0.05, **p < 0.01.

### 3.9 HFD and HRCD may induce specific differentially abundant *TCRβ CDR3* clonotypes that serve as signature sequences for the dietary interventions

After a series of the above characteristic analyses, we further investigated the differentially abundant *TCRβ CDR3* clonotypes between the two dietary intervention groups and the healthy Control group (**Figures 9A–D**). A total of 10 upregulated clonotypes in the thymus of the HFD group compared with the Control group (**Figure 9A**) and 10 upregulated clonotypes in the thymus of the HRCD group compared with the Control group (**Figure 9C**) were screened out; 8 upregulated clonotypes in the spleen of the HFD group relative to the Control group (**Figure 9B**) and 11 upregulated clonotypes in the spleen of the HRCD group relative to the Control group (**Figure 9D**) were identified. We identified the clonotype “*CSSRAGGLNSDYTF*”, whose expression was significantly higher in both the thymus and spleen of the HFD and HRCD groups relative to the Control group. The detailed information of the clonotypes in the figures is shown in **Supplementary Tables S1**-**S4**.

**Figure 9:**
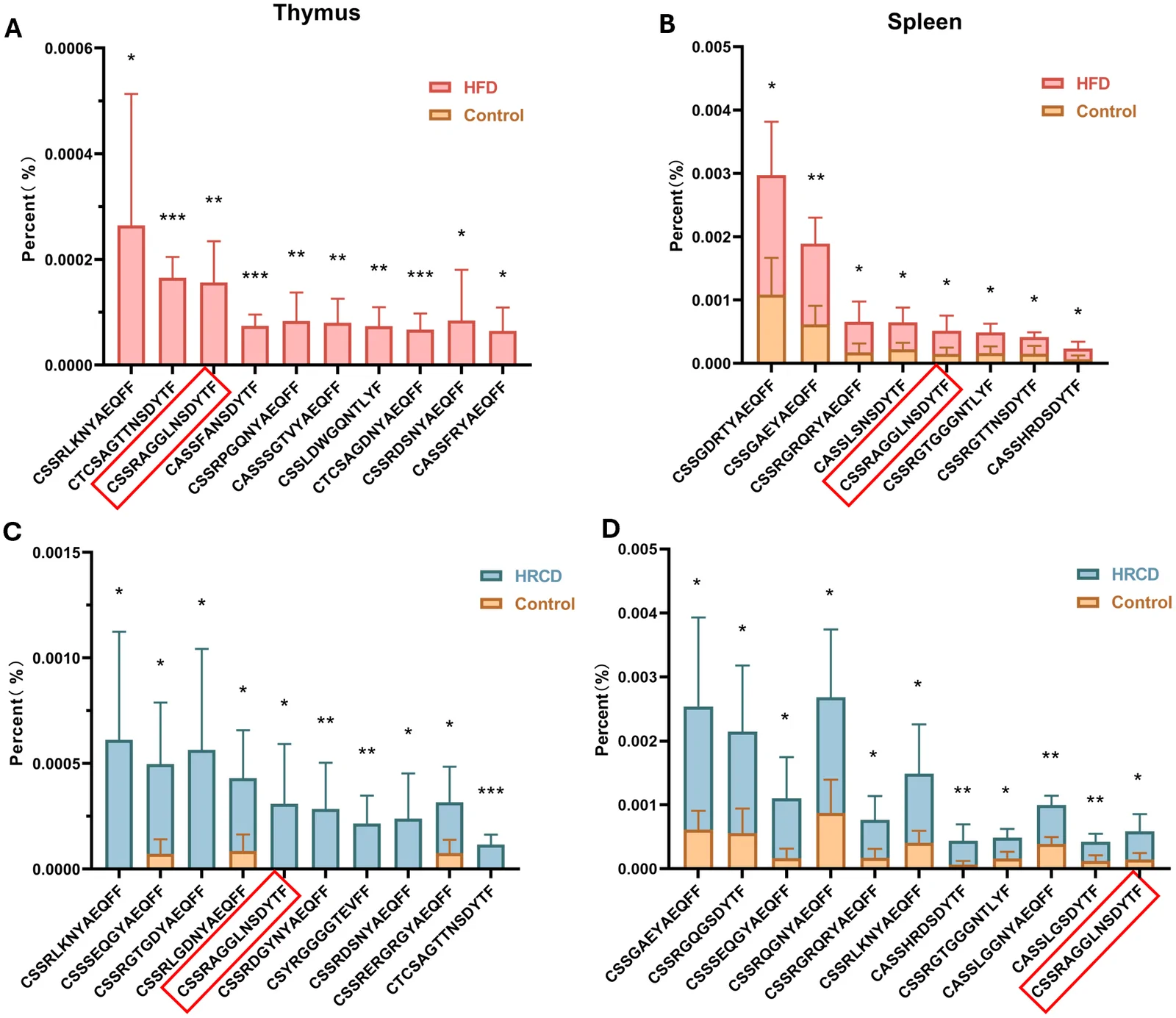
Differentially abundant CDR3 clonotypes in the *TCRβ CDR3* repertoire in the thymus and spleen of mice. The figure shows differentially abundant clonotypes in the thymus of HFD **(A)** and HRCD **(C)** groups compared to the Control group (if a clonotype is not shown for the Control group, it indicates the clonotype is absent in the Control group). Similarly, differentially abundant clonotypes in the spleen of HFD **(B)** and HRCD **(D)** groups relative to the Control group are presented (n=8). *p < 0.05, **p < 0.01, ***p <0.001.

## 4 Discussion

Our experiment explored the relevant changes in tissue morphology and molecular levels of central and peripheral immune organs in mice under the long-term influence of HFD and HRCD from young adulthood (3 months of age) to late middle age. We found that although both diets led to increased body weight, altered serum cytokine levels, immune-cell proportions in the thymus and spleen, as well as the characteristics of the *TCRβ CDR3* repertoire, with more prominent alterations observed in the spleen than in the thymus, the CDR3-related characteristics indicated that these immune alterations were mediated via distinct mechanisms by the two dietary patterns.

In the present study, mice in both the HFD and HRCD groups exhibited significantly lower food intake than the Control group, whereas average daily caloric intake was comparable among groups, largely ruling out excess caloric intake as a confounding variable that could induce alterations in the immune system of mice. Nonetheless, higher body weight persisted in the two diet groups, indicating an obese-like phenotype; thus, other obesity-related factors cannot be fully excluded. While serum total cholesterol (TC) and fasting blood glucose (FBG) were elevated in HFD and HRCD mice, triglyceride (TG) levels remained unchanged, suggesting TG is unlikely to mediate the observed immune alterations. Importantly, direct causality between elevated TC/FBG and immune remodeling cannot be established from our data, since metabolic and immune changes represent covariant downstream phenotypes induced by differences in dietary composition. Collectively, splenic immune alterations observed in this study are more likely attributable to dietary constituents rather than caloric intake, with diet composition may exerting more direct effects on immunometabolic outcomes.

Our experimental results revealed that both the HFD and HRCD groups triggered significant up-regulation of TNF-α (myeloid-derived inflammatory mediator), IL-10 (immunomodulatory factor) and FAS (tissue-damage- and apoptosis-associated protein). Nevertheless, predominant elevation of IL-6 (T-cell-associated factor) was observed in the HFD group, whereas the HRCD group showed dominant increases in IL-17A (Th17-cell-associated factor). Both dietary patterns may induce inflammatory-immunomodulatory-related pathways, though possibly with different priorities. Mice in both groups showed markedly increased serum pro-inflammatory cytokine levels, which may suggest pro-inflammatory immune activation; meanwhile, the anti-inflammatory immunomodulatory factor IL-10 was also substantially elevated, which may reflect a potential compensatory immunomodulatory response under inflammatory conditions (17). Collectively, these observations may point to a status where systemic inflammation coexists with immunomodulation.

Mice subjected to the two different dietary interventions exhibited divergent changes in cellular subsets within the thymus and spleen. In the thymus, the alterations in proportions of various immune-cell subsets were consistent between the two groups, with no significant changes observed. Mark et al. found that following 120-day HFD intervention, thymus weight and total thymocyte counts increased in mice, while the proportions of CD4^+^ and CD8^+^ T-cell subsets showed no obvious change (18). Our findings are in line with this report: no changes were observed in the proportions of all thymic immune-cell subsets in either the HFD or HRCD group. This phenomenon may occur because HFD and HRCD promote the proliferation of hematopoietic stem cells in the bone marrow and thymus without disrupting the core program of T-cell differentiation. Meanwhile, the relatively insulated thymic microenvironment shields it from direct impacts elicited by systemic metabolic shifts (19), thereby maintaining normal cellular composition (18). In contrast to the thymus, the spleen, as the body’s largest secondary lymphoid organ, is directly exposed to circulating inflammatory factors and displays greater sensitivity to immune perturbations (20). HFD and HRCD exerted marked differences in their effects on splenic immune-cell composition. HFD mainly affected T-cell, CD8^+^ T-cell and NKT-cell subsets, whose proportions were all significantly decreased; HRCD primarily triggered alterations in the DC subset, with a marked elevation in its proportion.

Notably, quantitative H&E histomorphometry revealed a significantly increased red-pulp-to-white-pulp (R/W) ratio and a marked reduction in white-pulp proportion in the HFD group, indicating remodeling of splenic compartmental structure under long-term high-fat-diet stress. Although the proportion of CD3^+^ T-cell-zone area measured by immunohistochemistry only exhibited a declining trend without statistical significance in both the HFD and HRCD groups, this result is partially reconcilable with the reduced splenic T-cell proportions observed by flow cytometry in the HFD group. This discrepancy arises because flow cytometry assesses T-cell abundance across the whole spleen, whereas immunohistochemistry evaluates T-cell spatial distribution within white-pulp from selected tissue sections. H&E staining depicts gross shifts in splenic red- and white-pulp compartments, while CD3 immunohistochemistry resolves intra-white-pulp T-cell distribution; together, these findings suggest that altered dietary composition may induce such splenic structural remodeling.

With respect to HFD, significantly elevated serum pro-inflammatory cytokines including TNF-α and IL-6 indicate a state of systemic chronic inflammation. Combined with increased FAS levels and the relative reduction in splenic white‑pulp proportion, this phenotype may be attributed to intracellular accumulation of long-chain saturated fatty acids within CD8^+^ T cells under HFD conditions, which directly impairs T-cell metabolism and function (21). Furthermore, HFD-mediated metabolic stress and inflammatory responses may reduce the proportions of CD8^+^ T cells and NKT cells by inducing their depletion or apoptosis (22). As for HRCD, the elevated proportion of splenic DCs may be associated with enhanced DC maturation and polarization toward a pro-inflammatory phenotype (23). In addition, the milder relative reduction in white‑pulp proportion, together with predominant upregulation of serum IL-17A rather than IL-6 in the HRCD group, also supports the existence of divergent inflammation-driving pathways between the two diets, which may partially explain their differential effects on distinct immune-cell subsets. Previous literature has documented synchronous reduction of T cells in peripheral blood and spleen of HFD-fed mice (24). Adipose tissue represents an important site for infiltration by CD8^+^ effector T cells (25), and iNKT cells are also depleted within adipose tissue (26). Accordingly, we speculate that immune cells decreased in the spleen may in part migrate to inflamed tissues such as adipose tissue to participate in local inflammatory responses or redistribute via the circulation. It should be noted that changes in splenic immune-cell proportions did not fully correspond to variations in serum cytokine levels in our experimental results. Since serum cytokines are derived from multiple tissues and organs across the whole body instead of being produced exclusively by splenic immune cells, there is no simple one-to-one relationship between these two sets of readouts. Moreover, splenic weight showed no significant difference in the HRCD group but increased significantly in the HFD group, further implying potential discrepancies in the status and magnitude of systemic inflammation induced by these two dietary patterns.

The present study suggests that both HFD and HRCD intervention may trigger the depletion of low-frequency clonal clonotypes and the selective expansion of intermediate-frequency clonal clonotypes in mouse splenic T cells, with a potentially more significant effect induced by HFD. Wyatt et al. reported that HFD intervention significantly increased the proportion of high-frequency CDR3 clonotypes and reduced that of low-frequency CDR3 clonotypes in CD8^+^ T cells isolated from mouse adipose tissue (27). This divergence between adipose tissue and spleen may reflect tissue-specific remodeling of the TCR clonal repertoire by HFD. Specifically, the spleen tends to exhibit a global loss of TCR diversity accompanied by expansion of intermediate-frequency clones, whereas adipose tissue is primarily marked by enrichment of high-frequency, locally pathogenic clones (7). Moreover, the emergence of numerous intermediate- and high-frequency clonal clonotypes in the spleen of mice is typically accompanied by a reduction in TCR diversity (28), a trend that appears to be in good agreement with the significant decrease in the splenic TCRβ CDR3 diversity index observed in the HFD group of our study. Although the diversity index in the HRCD group did not reach statistical significance, it also exhibited a declining trend, which may be largely consistent with the above-mentioned report. Collectively, these findings suggest that although the two dietary interventions may differ in their molecular mechanisms, they could induce highly similar patterns of TCR clonal repertoire remodeling at the level of clonal abundance distribution, characterized by depletion of low-frequency clonotypes and expansion of intermediate-frequency clonotypes (27, 29).

In terms of *TRBV/TRBJ* gene usage, the *V* gene usage in the thymus appeared similar between the HFD and HRCD groups, and both groups showed preferential usage of *V12–1* in the spleen. However, HRCD was associated with significantly fewer changes in *V* and *J* gene usage compared with HFD. In addition to the previously reported preferential usage of the *V12* family in the spleen in HFD mice (7), we further observed preferential usage of *V23* in the spleen, as well as preferential usage of *V12-1* and *V5* in the thymus of HFD mice. The usage of *TRBV* genes is intrinsically non-random. Their rearrangement efficiency may exhibit inherent bias shaped by the quality of recombination signal sequences and the positional context of gene loci, which could be further molded by thymic positive and negative selection, ultimately generating tissue-specific patterns of *V*-gene usage (30, 31). Therefore, the altered *V*-gene usage observed in the present study may reflect the combined effects of intrinsic rearrangement bias, thymic selection, and the pressure of peripheral clonal expansion. Previous studies have reported that dietary intervention can trigger perturbations in the thymic TCR repertoire and constrain TCR diversity (32). Moreover, T-cells derived from adipose tissue may display substantial loss of diversity across multiple *Vβ* gene families (7). Antigen-driven restriction in the usage of TCR *V*-gene families has also been noted among adipose-tissue T cells from HFD-fed mice (33), suggesting that biased *V*-gene usage under a diet-associated systemic inflammatory milieu may be linked to antigen- or inflammation-driven clonal selection. Consistent with this trend, reduced usage of *TRBV19*, *TRBV2* and *TRBV4* together with elevated usage of *TRBV12-1* and *TRBV23* was detected in the spleen of the HFD group in our study, which may reflect the depletion of clones corresponding to certain *V* genes and the expansion of specific clones under inflammatory conditions. Meanwhile, *TRBV12-1* showed increased usage in both the thymus and spleen of mice from the HFD and HRCD groups, with concordant directional changes across both diets and organs. This may indicate its potential association with clonal expansion or selective preference linked to dietary responses, which merits further attention. Notably, *J* genes are generally considered to be highly stable and conserved (30). In our study, HFD mice displayed significant differences in multiple *J*-gene usages in the spleen, whereas only *J1-5* appeared altered in the thymus. By contrast, HRCD mice showed significant changes in multiple *J*-gene usages in the thymus, with no detectable differences in the spleen. Such opposing observations warrant further investigation.

Different *J* gene usage may correspond to relevant changes in CDR3 length (34). Shorter CDR3 clonotypes form tighter and more stable immune synapses with stronger TCR signal transduction intensity; T cells harboring shorter CDR3 display higher efficiency in initiating acute immune responses. By contrast, longer CDR3 clonotypes form loose and unstable immunological synapses with weaker TCR signaling strength. In our study, neither HFD nor HRCD altered the peak of CDR3 length distribution, which remained a bell-shaped distribution centered on 14 and 15 amino acids, suggesting that the overall architecture of the TCR repertoire was largely conserved. However, splenic T cells from the HFD group exhibited a length shift characterized by a decreased proportion of short CDR3 clonotypes and an increased proportion of long CDR3 clonotypes. This observation is consistent with previous reports demonstrating HFD-driven TCR clonal expansion and selective shifts in CDR3 properties in mice (27). Furthermore, CDR3 length has been reported to correlate with specific murine Vβ–Jβ segment combinations (35), indicating that CDR3 length distribution may be influenced by gene usage during TCR rearrangement. Changes in the thymus were characterized by an increased proportion of short CDR3 clonotypes (CDR3 of 11 amino acids in the HFD group and CDR3 of 8 and 9 amino acids in the HRCD group), with no obvious alteration in the proportion of long CDR3 clonotypes. These findings suggest that both diets may shift the thymic repertoire toward short CDR3 clonotypes by affecting TCR rearrangement or selection within the thymus. Given the relatively insulated thymic microenvironment, the scope of these alterations is more restricted than that observed in the spleen. This is plausibly attributed to the HFD-induced inflammatory microenvironment which can weaken the normal restrictive effect of MHC molecules on CDR3 length, allowing long CDR3 T cells to survive and expand, which are difficult to survive under physiological conditions (36). However, in the HRCD group, only the proportions of a small subset of short CDR3 clonotypes were altered in the thymus and spleen, while no changes were observed for long CDR3 clonotypes. This pattern differs from that of the HFD group, which is characterized by shifts in CDR3 length, further suggesting that the two dietary regimens modulate the T-cell receptor repertoire via distinct mechanisms; the specific underlying mechanisms require further verification.

In the comparison of Jaccard index, both HFD and HRCD may induce the generation of certain shared high-frequency CDR3 clonotypes in the mouse thymus, which may make their Jaccard index significantly higher than that of the Control group. The splenic *TCRβ CDR3* repertoire tends to exhibit high individual specificity and strong natural heterogeneity; thus, although the HFD group appeared to show a decreasing trend in similarity, no statistically significant difference was observed due to large intra-group variation, which may be consistent with previous research conclusions (37, 38). In addition, all differentially abundant CDR3 clonotypes shown in **Figure 9** are reported for the first time in the present study. During the screening process, we identified the clonotype “CSSRAGGLNSDYTF”, whose expression was significantly higher in both the thymus and spleen of the HFD and HRCD groups relative to the Control group. No relevant literature has been reported so far; its specific function requires subsequent verification and merits focused attention.

## 5 Conclusion

This study performed long-term HFD and HRCD intervention in male C57BL/6J mice starting at 3 months of age until 14 months of age. After systematic analysis of multiple immune-related parameters, it was found that HFD and HRCD may trigger immune disorders through distinct molecular mechanisms. This study provides experimental evidence for dissecting the regulatory effects of dietary composition on the immune system and the pathogenesis of immune disorders induced by HFD and HRCD, and also identifies potential biomarkers.

## 6 Limitations

The present study has a limitation that only male mice were used. Previous studies have demonstrated sex differences in the immune responses to dietary interventions. In HFD-induced obesity models, female mice are protected against systemic low-grade inflammation, whereas males are more prone to adipose tissue inflammation and alterations in immune-cell composition (39). A systematic review including 41 studies also confirmed that female rodents exhibit lower levels of systemic inflammation than males in diet-induced obesity models (40). Furthermore, adipose tissue Tregs display sexual dimorphism, and sex hormone signaling can regulate their clonal expansion and TCR repertoire characteristics (41). Therefore, the immunological conclusions drawn from male mice in this study cannot be directly extrapolated to female animals.

## Data Availability

The datasets presented in this study can be found in online repositories. The names of the repository/repositories and accession number(s) can be found below: https://www.ncbi.nlm.nih.gov/, PRJNA1437728.
