# Supplementary material for: Long-term high-fat diet and long-term high-refined-carbohydrate diet induce alterations in the splenic TCRβ CDR3 repertoire and peripheral multi-compartmental immune parameters in mice
Source: Front Immunol. 2026 Oct 14;17:1947535. doi: 10.3389/fimmu.2026.1947535 (PMC13713846; doi:10.3389/fimmu.2026.1947535)
Supplement: Supplementary file 1 [file DataSheet1.docx]

Supplementary Material

## 1. Supplementary Figures

**Supplementary Figure 1.** *TRBV* gene usage in individual thymic samples.

**Supplementary Figure 2.** *TRBJ* gene usage in individual thymic samples.

**Supplementary Figure 3.** *TRBV* gene usage in individual splenic samples.

**Supplementary Figure 4.** *TRBJ* gene usage in individual splenic samples.

**Supplementary Figure 5.** *TRBV-TRBJ* paired usage in the thymus and spleen of the Control, HFD and HRCD groups.

## 2. Supplementary Tables

**Supplementary Table 1.** Top 10 sequences with significantly higher expression in the thymus of the HRCD group than the control group

|  | *TRBV* | *TRBJ* | CDR3 Sequence | p |
| --- | --- | --- | --- | --- |
| 1 | 30 | 2-1 | CSSRLKNYAEQFF | 0.012 |
| 2 | 30 | 2-1 | CSSSEQGYAEQFF | 0.011 |
| 3 | 30 | 2-1 | CSSRGTGDYAEQFF | 0.012 |
| 4 | 30 | 2-1 | CSSRLGDNYAEQFF | 0.014 |
| 5 | 30 | 1-2 | CSSRAGGLNSDYTF | 0.017 |
| 6 | 30 | 2-1 | CSSRDGYNYAEQFF | 0.008 |
| 7 | 30 | 1-1 | CSYRGGGGTEVFF | 0.003 |
| 8 | 30 | 2-1 | CSSRDSNYAEQFF | 0.016 |
| 9 | 30 | 2-1 | CSSRERGRGYAEQFF | 0.03 |
| 10 | 1 | 1-2 | CTCSAGTTNSDYTF | 0.0003 |

**Supplementary Table 2.** Top 10 sequences with significantly higher expression in the thymus of the HFD group than the control group

|  | *TRBV* | *TRBJ* | CDR3 Sequence | p |
| --- | --- | --- | --- | --- |
| 1 | 30 | 2-1 | CSSRLKNYAEQFF | 0.020 |
| 2 | 1 | 1-2 | CTCSAGTTNSDYTF | 0.00007 |
| 3 | 30 | 1-2 | CSSRAGGLNSDYTF | 0.001 |
| 4 | 14 | 1-2 | CASSFANSDYTF | 0.000024 |
| 5 | 30 | 2-1 | CSSRPGQNYAEQFF | 0.003 |
| 6 | 14 | 2-1 | CASSSGTVYAEQFF | 0.002 |
| 7 | 30 | 2-4 | CSSLDWGQNTLYF | 0.001 |
| 8 | 1 | 2-1 | CTCSAGDNYAEQFF | 0.000457 |
| 9 | 30 | 2-1 | CSSRDSNYAEQFF | 0.042 |
| 10 | 14 | 2-1 | CASSFRYAEQFF | 0.04 |

**Supplementary Table 3.** Top 11 sequences with significantly higher expression in the spleen of the HRCD group than the control group

|  | *TRBV* | *TRBJ* | CDR3 Sequence | p |
| --- | --- | --- | --- | --- |
| 1 | 30 | 2-1 | CSSGAEYAEQFF | 0.033 |
| 2 | 30 | 1-2 | CSSRGQGSDYTF | 0.027 |
| 3 | 30 | 2-1 | CSSSEQGYAEQFF | 0.012 |
| 4 | 30 | 2-1 | CSSRQGNYAEQFF | 0.049 |
| 5 | 30 | 2-1 | CSSRGRQRYAEQFF | 0.015 |
| 6 | 30 | 2-1 | CSSRLKNYAEQFF | 0.045 |
| 7 | 14 | 1-2 | CASSHRDSDYTF | 0.005 |
| 8 | 30 | 1-3 | CSSRGTGGGNTLYF | 0.018 |
| 9 | 14 | 2-1 | CASSLGGNYAEQFF | 0.004 |
| 10 | 29 | 1-2 | CASSLGSDYTF | 0.006 |
| 11 | 30 | 1-2 | CSSRAGGLNSDYTF | 0.018 |

**Supplementary Table 4.** Top 8 sequences with significantly higher expression in the spleen of the HFD group than the control group

|  | *TRBV* | *TRBJ* | CDR3 Sequence | p |
| --- | --- | --- | --- | --- |
| 1 | 30 | 2-1 | CSSRLKNYAEQFF | 0.043 |
| 2 | 30 | 2-1 | CSSSEQGYAEQFF | 0.002 |
| 3 | 30 | 2-1 | CSSRGTGDYAEQFF | 0.025 |
| 4 | 14 | 1-2 | CSSRLGDNYAEQFF | 0.048 |
| 5 | 30 | 1-2 | CSSRAGGLNSDYTF | 0.038 |
| 6 | 30 | 1-3 | CSSRDGYNYAEQFF | 0.018 |
| 7 | 30 | 1-2 | CSYRGGGGTEVFF | 0.049 |
| 8 | 14 | 1-2 | CSSRDSNYAEQFF | 0.034 |
